# Understanding the Conductive Carbon Additive on Electrode/Electrolyte Interface Formation in Lithium-Ion Batteries via *in situ* Scanning Electrochemical Microscopy

**DOI:** 10.3389/fchem.2020.00114

**Published:** 2020-02-25

**Authors:** Shuai Liu, Xiaojie Zeng, Dongqing Liu, Shuwei Wang, Lihan Zhang, Rui Zhao, Feiyu Kang, Baohua Li

**Affiliations:** ^1^Shenzhen Key Laboratory on Power Battery Safety Research and Shenzhen Geim Graphene Center, Tsinghua Shenzhen International Graduate School, Shenzhen, China; ^2^Laboratory of Advanced Materials, School of Materials Science and Engineering, Tsinghua University, Beijing, China

**Keywords:** conductive carbon additive, electrode/electrolyte interface, lithium-ion batteries, scanning electrochemical microscopy, solid electrolyte interface (SEI)

## Abstract

The role of conductive carbon additive on the electrode/electrolyte interface formation mechanism was examined in the low-potential (3.0–0 V) and high-potential (3.0–4.7 V) regions. Here the most commonly used conductive carbon Super P was used to prepared electrode with polyvinylidene fluoride binder without any active material. The dynamic process of interface formation was observed with *in situ* Scanning Electrochemical Microscopy. The electronically insulating electrode/electrolyte passivation layer with areal heterogeneity was formed after cycles in both potential regions. The low-potential interface layer is mainly composed of inorganic compounds covering the conductive carbon surface; While the electrode after high-potential sweep tends to lose its original carbon structure and has more organic species formed on its surface.

## Introduction

Lithium-ion batteries (LIBs) are one of the most commonly used energy storage devices and have been used in portable electronics devices and electric vehicles. During the (dis)charge reactions, interfacial reactions take place between the electrode and electrolyte to form the electrode/electrolyte interface (EEI), which is crucial for cell performance (Gauthier et al., 2015; Tripathi et al., 2018). In the composite electrode, the conductive carbon additive is added to improve the electrical conductivity of active materials and was once considered as an “inactive” component. Recently, the high surface reactivity of conductive carbon on EEI formation has been revealed gradually (Hou et al., 2017; Li and Manthiram, 2019).

In the composite electrode, even though the weight percent of conductive additive is low, it has high surface area and atomic percentage to cover most of the electrode surface (Younesi et al., 2015). In addition, the carbonaceous materials have various chemical functional groups on its surface, including hydroxyl-, carboxyl-, carbonyl-, and aromatic groups. The large surface area and various functional groups of conductive carbon react with electrolyte to form the EEI both spontaneously and during electrochemical cycling. During the spontaneous reaction, the carbon additive interacts with electrolyte through corrosion-like reactions. The spontaneous polymerization of solvent molecules form the EEI with similar composition as the EEI after electrochemical cycling (Membreño et al., 2015). This may partially explain the detection of similar degradation products on the negative and positive electrodes even though different reactions (reduction and oxidation) are involved (Gauthier et al., 2015). When cycling in the low-potential region, the conductive carbon has lithium storage property but shows large irreversible capacity through the formation of the solid electrolyte interface (SEI) (Fransson et al., 2001; Gnanamuthu and Lee, 2011; Anothumakkool et al., 2018). In the high-potential region, the spontaneously formed EEI will be partially decomposed via oxidization or desorption when charged to 4.3–4.5V (Younesi et al., 2015). The passivation role of carbon black for the Ni-rich electrodes has been considered between 3.0V and 4.5V, suggesting that the organic complexes generated on carbon migrate across the active material's surface to suppress the unwanted interfacial reactions to certain extent (Li et al., 2017a). In addition, the dynamic evolution of interface by the mass transfer between carbon black and active material was observed (Li and Manthiram, 2019); However, the passivation effect of conductive carbon lose stability at extreme potentials (>4.5 V), which is mainly due to (de)intercalation of anions, irreversible electrolyte oxidation and degradation of conductive carbon (Zheng et al., 2013; Li et al., 2014; Qi et al., 2014; Kajiyama et al., 2015; Metzger et al., 2015; Younesi et al., 2015; He et al., 2016; Scipioni et al., 2016).

In the composite electrode, the contribution of conductive carbon to interfacial composition is equivalent or larger than the contribution of the active material. Therefore, the reactivity of conductive carbon, and its influence on the EEI formation once considered “inactive” toward electrolytes, should be reconsidered. However, most of the EEI studies were derived from composite electrodes that contain active material, carbon additive and binder, which leads to an ambiguous interpretation of the individual components for the EEI formation. This calls for the investigation of individual cell component and its effect on the interfacial changes (Li et al., 2017b). In addition, the application of *in situ/operando* technique is necessary to capture the dynamic formation of EEI (Gauthier et al., 2015; Wang et al., 2018; Liu et al., 2019a).

In the present study, the reactivity of conductive carbon additives on the EEI was investigated individually to give a better understanding of its contribution on interface dynamics. The interface formation process was examined in the low-potential and high-potential regions, with the EEI termed as solid electrolyte interface (SEI) and cathode electrolyte interface (CEI), respectively. The electrode was prepared with the commonly used conductive carbon additive Super P and binder polyvinylidene fluoride (PVDF) without any active material (Zheng et al., 2013; Li et al., 2014; Qi et al., 2014; Kajiyama et al., 2015; Metzger et al., 2015; Younesi et al., 2015). The dynamic evolution of the interfaces was observed with *in situ* Scanning Electrochemical Microscopy (SECM). SECM is a four-electrode electroanalytical scanning probe technique and has been applied for the interface study of Li-ion batteries in recent years (Polcari et al., 2016; Liu et al., 2019a). The feedback mode of SECM is commonly used for interface study (Zampardi et al., 2013; Heinz et al., 2014; Liu et al., 2019b). During the measurement, the scanning probe detected the electrochemical reactivity of electrode substrate through the regeneration rate of redox mediator from the substrate. It is understood that the conductivity of the electrode will decrease with the SEI/CEI passivation layer formed on the surface. By using the feedback curve and feedback image of SECM, the reactivity of the electrode substrate and interfacial dynamic change with cycles could be revealed.

## Experimental Section

The electrode was prepared by mixing equal amounts of conductive carbon Super P (TIMCAL) and binder PVDF (Arkema) (1:1 by wt.%) dissolved in N-methyl pyrrolidone (NMP) to obtain a uniform slurry. Then the slurry was deposited on to the SECM substrate with glass carbon as current collector and dried at 80°C overnight. The electrolyte used was 1 M LiPF_6_ in EC:DMC:DEC (1:1:1 vol %) (CAPCHEM) containing 10 mM ferrocene (Macklin, 99%) as redox mediator. All the electrochemical cyclic voltammetry (CV) tests and SECM measurement were performed using Bio-Logic M470 in the Ar-filled glovebox (MIKROUNA). The experiment setup consists of a four-electrode open cell as shown in detail in our previous publication (Liu et al., 2019c). One working electrode is the electrode pasted substrate, the other working electrode is the 15 μm Pt microprobe sealed in glass (RG = 10), the counter electrode and reference electrode were all made of Li foils. The substrate electrode was cycled at a scan rate of 5 mV/s in the potential windows of 3.0–0.0 V and 3.0–4.7 V, referred to as the low-potential region and high-potential region, respectively. Prior to SECM measurement, the substrate was tilt corrected with a gradienter, and then the microprobe was loaded at the vicinity of the electrode. The approach curve was recorded with step size of 2 μm and 0.5 μm when the feedback change was larger than 115% or smaller than 75%. The area scan was recorded with the tip near the substrate surface, at a step size of 8 μm for the 120 μm × 120 μm area. A Scanning Electron Microscope (SEM, HITACHI, SU8010) was used to characterize the morphology information. X-ray Photoelectron Spectroscopy (XPS, PHI5000VersaProbeII) using monochromatic Al Kα radiation (1486.6 eV) with depth profiling was used to analyze the chemical composition of electrode surface. The microscopic structure of the electrode surface was further analyzed by transmission electron microscopy (TEM, FEI Tecnai G2 F30). Prior to the material characterizations, the cycled electrodes were all rinsed with dimethyl carbonate (DMC) in the glovebox.

## Results and Discussion

### The Feedback Mode of SECM

**Figure 1:**
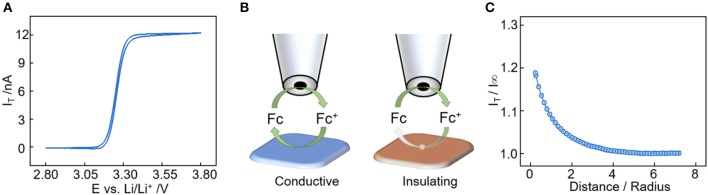
**(A)** Cyclic voltammetry of the SECM probe in 1 M LiPF_6_ in EC:DMC:DEC (1:1:1 by vol.%) electrolyte with 10 mM ferrocene at scan rate of 10 mV/s. **(B)** SECM feedback mode over an conductive substrate (positive feedback) and insulating substrate (negative feedback). **(C)** SECM approach curve toward the Super P electrode at step size of 2 μm.

The feedback mode of SECM is frequently used for the interface evolution study in LIBs (Liu et al., 2019a). In this mode, the microprobe monitors the regeneration rate of the redox mediator from the substrate to determine its electronic character. Ferrocene was chosen as the free-diffusion redox mediator and the corresponding cyclic voltammetry between 2.8 and 3.8 V is shown in Figure 1A. The redox potential of the ferrocene/ferrocenium (Fc/Fc^+^) combination is 3.26 V vs. Li^+^/Li, which is suitable for solid electrolyte interface characterization in LIBs (Zampardi et al., 2013; Ventosa et al., 2017). During the feedback measurement (Figure 1B), the microprobe has an applied potential of 3.6 V vs. Li^+^/Li as it moves toward the substrate, and the current at the microprobe increases when the substrate is conductive due to the increase of Fc regeneration rate (positive feedback). The microprobe current decreases when the substrate is passivated because the regeneration and diffusion of Fc are all hindered by the insulating substrate (negative feedback). In Figure 1C, a positive feedback is observed when approaching the pristine electrode surface, indicating the conductive nature of the carbon additive electrode. SEM pictures of Super P powder and electrode are shown in Figure S1, the Super P powder has sub-hundred nanometer spherical shaped particles, and the morphology doesn't change much after being made into an electrode. In addition, the Super P has high surface area of 63.41 m^2^ g^−1^ from the BET measurement result.

### EEI Formation via *in situ* SECM in the Low-Potential Region

**Figure 2:**
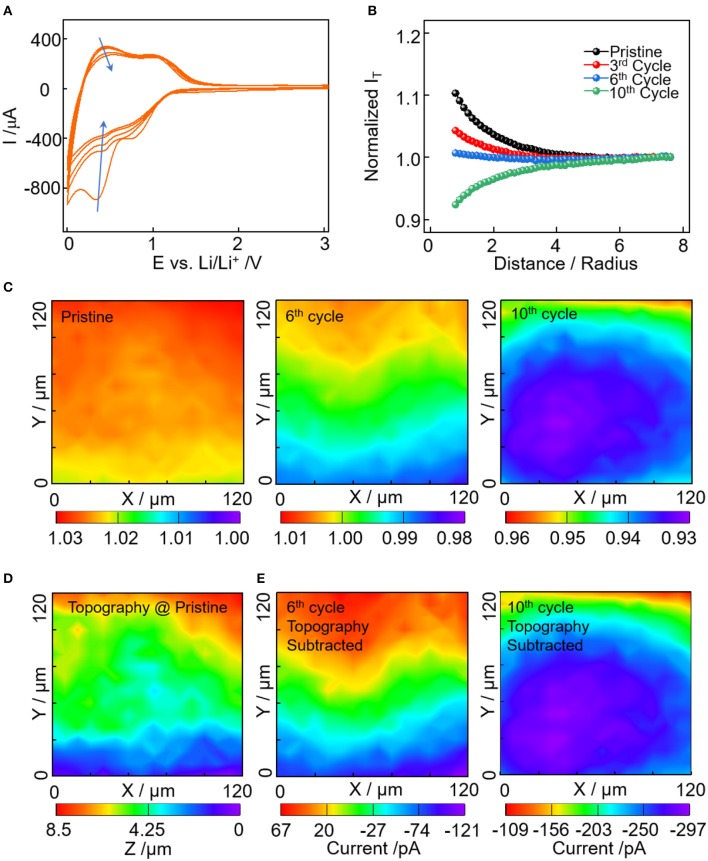
**(A)** Cyclic voltammetry of Super P electrode from 3.0 to 0 V vs. Li^+^/Li in electrolyte of 1M LiPF_6_ in EC:DMC:DEC at a scan rate of 5 mV/s. **(B)** Normalized approach curves toward electrode at pristine, after 3rd, 6th, and 10th cycles. **(C)** SECM area scan with normalized current density of the electrode at pristine, after 6 and 10th cycles. **(D)** SECM feedback current converted topography of the scanned area. **(E)** SECM area scan with the topography induced feedback current subtracted for the 6 and 10th cycle.

To investigate the effect of conductive carbon on the EEI formation, the approach curve and area scan of SECM were employed to examine the change of substrate conductivity. Figure 2A shows the first five cycles of Super P electrode ranged between 3.0 and 0.0 V vs. Li^+^/Li. Two characteristic reversible capacity regions were observed, one in 1.4–0.6V and the other below 0.6V. This is ascribed to the specific lithium storage mechanism of Super P, i.e., in the vicinity of turbostratic graphene edges (Anothumakkool et al., 2018). In addition, the irreversible capacity is high especially for the first few cycles, which is caused by the electrolyte reduction and SEI formation (Fransson et al., 2001; Gnanamuthu and Lee, 2011). In Figure 2B, the approach curves were recorded over the electrodes at a pristine state and after cycles. The approach curves change from positive feedback to negative feedback, and the normalized current densities decrease with cycles, indicating the kinetics for the Fc^+^ reduction get slower on the electrode with SEI formation. Figure 2C shows the corresponding area scan of an identical 120 × 120 μm region with cycles. For a better comparison, the different sets of area scan were all plotted with the normalized current densities varying <0.03. At pristine state, the normalized current densities are larger than 1.02, indicating the good conductivity of the electrode substrate. In addition, it's commonly assumed that the pristine electrode is uniformly distributed and the variation in the feedback current density is solely caused by topographical difference (Heinz et al., 2014; Liu et al., 2019b,c,d). Thus, the area scan of the pristine electrode in Figure 2C was converted to topographical information via calculation with the approach curve (Figure S2). In Figure 2D, the scanned area has a topographical variation of ~8.5 μm, corresponding to the increase of feedback current with substrate height (Figure 2C).

With the cycling process, SEI evolves and thus the feedback images are not only reflecting the topography but also the lateral heterogeneity of SEI (Heinz et al., 2014). Since the thickness of SEI are in the range of 20~50 nm, the thickness can't induce feedback current change larger than 1 pA, which is negligible compared to the ~nA scale feedback image. So the feedback image in Figure 2C (6 and 10th cycles) are a result of the pristine topographical difference and the heterogeneous passivating properties of SEI for the scanned area. With the pristine topography (Figure 2D), the evolution of passivating SEI can be determined via calculation in combination with their corresponding approach curves (detailed calculation in Supplementary Materials). In Figure 2E, the heterogenous passivating SEI can be seen more clearly with the pristine topography subtracted. For the 6th cycle in Figure 2E, the feedback currents change from 70 to −120 pA as the image changes from red to purple, indicating the decrease of the mediator regeneration rate with SEI passivation. In accordance with the topography (Figure 2D), the passivation layer tends to form in the y <70 μm region. But the SEI passivation sites do not necessarily correlate with the substrate height. This can be seen clearly from the identical height region (Figure 2D: 20 μm < y <110 μm), which has the mixture of positive and negative feedbacks (Figure 2E). Upon the 10th cycle, the feedback current drops to the range of −100 ~ −300 pA, indicating a much slower mediator regeneration rate and more severe SEI passivation effect. Compared with the 6th cycle, the passivation region expands to a larger area with an elliptical shape covering the majority of the scanned area. It demonstrate that the passivating SEI grows with cycles and the growth has lateral heterogeneous properties.

### EEI Formation via *in situ* SECM in the High-Potential Region

**Figure 3:**
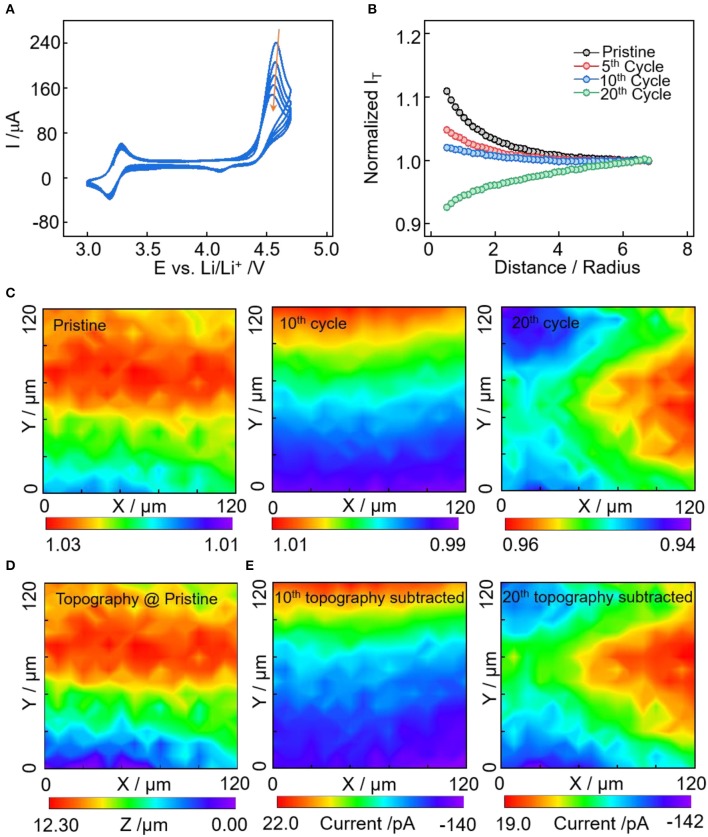
**(A)** Cyclic voltammetry of Super P electrode from 3.0 to 4.7 V vs. Li^+^/Li in electrolyte of 1 M LiPF_6_ in EC:DMC:DEC at a scan rate of 5 mV/s. **(B)** Normalized approach curves toward electrodes at pristine, after 5, 10, and 20th cycles. **(C)** SECM area scan with normalized current density of electrodes at pristine, and after 10 and 20th cycles. **(D)** SECM feedback current converted topography of the scanned area. **(E)** SECM area scan with the topography feedback current subtracted for the 10 and 20th cycle.

The conductive carbon electrode is cycled between 3.0 and 4.7 V vs. Li^+^/Li to examine the interface evolution in the high-potential region. In Figure 3A of the cyclic voltammetry curves, a pair of reversible redox peaks were observed at 3.28/3.18 V, corresponding to the oxidation and reduction reactions of ferrocene. In addition, the redox peaks at 4.56/4.10 V have obvious larger anodic peak than the cathodic peak, demonstrating the existence of an irreversible oxidation process. The anodic peak can be induced by anion intercalation, oxidative decomposition of electrolyte and conductive carbon oxidation (itself or its functional groups) (Qi et al., 2014, 2015; Soon et al., 2018). With these three contributions, the reversible part could be the (de)intercalation of ${\text{PF}}_{6}^{-}$ anions to/from the electrodes (Qi et al., 2014). The oxidation of electrolyte on conductive carbon at potential larger than 4.6 V is irreversible once the cathode electrolyte interface has formed (La Mantia et al., 2013; Li et al., 2013, 2014). The interface layer prefers to form on the carbon additive of the electrodes (Nordh et al., 2015). In addition, carbon black degrades with cycling via agglomeration and loss of crystallinity to amorphous structure. This leads to the decrease of the electrical conductivity, increase in the heterogeneity of the conductive carbon network, and reduction in the electron percolation of the composite electrode (Ngo et al., 2016; Scipioni et al., 2016).

The change of electronic character of the electrode substrate with cycles was examined by the approach curve and area scan of SECM (Figures 3B,C). In accordance with the result of low-potential sweep (Figure 2B), the approach curves change from positive feedback to negative feedback with cycles, indicating the decrease of electrode substrate conductivity induced by electrolyte decomposition and conductive carbon degradation. At the same sweep rate of CV, the passivation rate of the interface layer in the high-potential region (3.0–4.7V) is slower than that in the low-potential region (3.0–0.0V), which is caused by the different interface components. SECM area scan was applied to visualize the areal heterogeneity of interface evolution (Figure 3C), here we also converted the pristine feedback image to topographical information (Figure 3D), and the area scan after cycles having the topography feedback subtracted, i.e., passivation property feedback (Figure 3E). In Figure 3D, the scanned area has a topographical variation of ~12 μm with higher upper region (60 < y <100). After the 10th cycle, the passivation layer covers the majority of the electrode surface with negative feedback current densities, which is induced by electrolyte decomposition and conductive carbon degradation. Upon the 20th cycle, the magnitude of areal feedback current is not decreased, while the areal heterogeneity of the passivation layer is becoming more obvious. The nearly unchanged areal feedback current magnitude from the 10 to 20th cycles is unexpected. This could because the surface of conductive carbon has been covered by the electrolyte degradation products in the first ten cycles; for the second ten cycles, when a large amount of the surface area and active functional groups of conductive carbon has been covered, the oxidative decomposition of electrolytes is less severe. While the structural degradation, aggregation, and agglomeration of conductive carbon last throughout the whole cycling process, which may leads to the evolution of passivation layer and increase of areal heterogeneity.

### Morphology, Chemical and Structure Information of EEI

**Figure 4:**
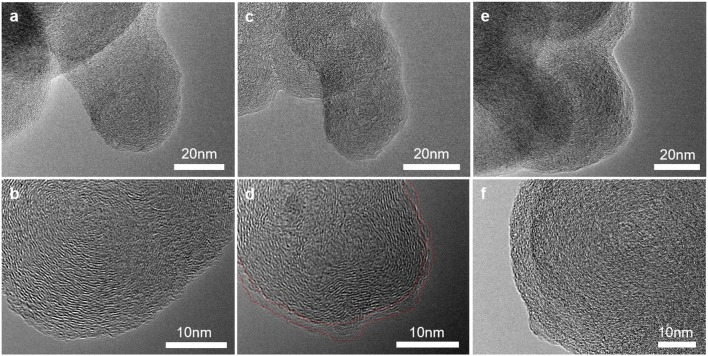
Transmission electron microscopy images of carbon black electrode **(a,b)** at pristine state, **(c,d)** after cycles in the low-potential and **(e,f)** high-potential regions.

TEM was applied to investigate the morphology and microstructure of the pristine and cycled conductive carbon electrodes at nanoscale. At pristine state in Figures 4a,b, the Super P particles have spherical shape with diameter of 30–50 nm. The particles have quasi-crystal structure and the graphitic structures are oriented concentrically tangent toward the surface of the primary particles (Gnanamuthu and Lee, 2011). After lithiation, the internal structure of the Super P particles were preserved while ~5 nm thick SEI layer is formed on the particle surface (Figures 4c,d) (Anothumakkool et al., 2018). In Figures 4e,f, the particles of the charged sample swelled and lost crystallinity. The structural degradation can be partially explained by the anion intercalation and co-intercalation of solvent molecules to expand the interlayer and electrolyte decomposition inside the carbon (Qi et al., 2014).

**Figure 5:**
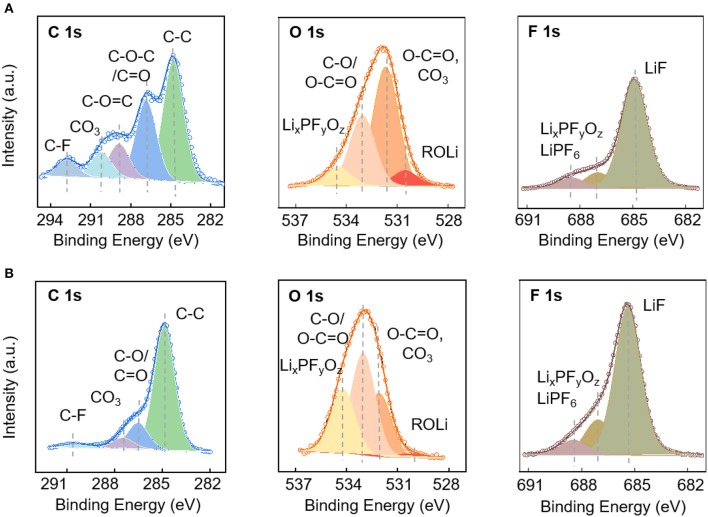
XPS characterization of electrodes: C1s, O1s, and F1s spectra of the electrodes cycled in the potential window of 0–3 V **(A)** and 3.0–4.7 V **(B)**.

The electrodes after cycles in the low-potential and high-potential regions were analyzed by XPS to provide information about the chemical composition on the surface. The elemental concentration of major components are listed in Table 1 with the corresponding C1s, O1s, and F1s spectra shown in Figure 5. The chemical composition of the solid electrolyte interfaces cycled in the low-potential and high-potential windows are similar except for the difference in the composition amount. This might start from the spontaneous reaction between the carbonate electrode and electrolyte after immersion in the electrolyte. Membreño et al. revealed the spontaneous decomposition of electrolyte on the carbon additive in a corrosion-like reaction with spontaneous polymerization reactions on the carbon surface (Membreño et al., 2015). The functional groups include alkanes (C-C), alkoxides and ethers (RCO), lithium carboxylate and esters (ROCO), carbonates (RCO_3_), fluoroalkanes (RCF_n_), lithium fluoride (LiF) and degraded lithium salt products (Li_x_PF_y_ and Li_x_PO_y_F_z_) (Qi et al., 2015; Younesi et al., 2015). The spontaneously formed SEI evolves during the electrochemical cycling in the low and high potential regions. After cycles in the 0–3 V vs. Li^+^/Li, the electrode is covered by a large fraction of inorganic compounds, LiF (mostly), Li_2_CO_3_, and Li_x_PF_y_O_z_, and fewer organic species (RCO, ROCO, ROCO_2_, and RCO_3_) formed via the degradation of salt and solvents. In comparison, the electrode cycled in the high-potential region has fewer inorganic species, which can be told from the lower amount of Li1s, F1s, and much higher C1s element concentration (Table 1). In addition, the much higher ratio between C-C bonds and carbon oxygen bonds demonstrates that the electrode surface has less oxygen-containing species and less electrolyte decomposition (Soon et al., 2018; Tatara et al., 2019).

**Table 1:** Element concentration on the surface of electrodes after cycling in the low-potential and high-potential regions.

**Electrodes**	**C1s**	**O1s**	**F1s**	**Li1s**
0–3 V	9.35	7.36	38.45	44.85
3–4.7V	30.02	6.33	31.54	31.76

## Conclusion

The influence of conductive carbon Super P on the electrode/electrolyte interface formation mechanism has been investigated by *in situ* SECM in combination with *ex situ* SEM, TEM, and XPS. After cycles in the low-potential region (3.0–0 V), the interface is covered by a passivation layer composed of salt and solvent degradation components. The interface layer grows with cycles and has lateral heterogenous properties. In the high-potential region (3.0–4.7 V), a similar passivation layer is formed while the passivation effect is relatively weaker with more organic species formed on the surface. In addition to the oxidative decomposition of electrolyte, the conductive carbon oxidation and anion intercalation lead to the degradation of conductive carbon itself. The EEI study with conductive carbon electrode helps to give a better understanding of its contribution to the interface dynamics in the composite electrode.

## Data Availability Statement

All datasets generated for this study are included in the article/Supplementary Material.

## Author Contributions

SL and XZ designed and finished the SECM experiment. DL organized the data and finalized the paper. SW, LZ, and RZ helped for the material characterization. FK and BL provided the experiment platform and funding support.

### Conflict of Interest

The authors declare that the research was conducted in the absence of any commercial or financial relationships that could be construed as a potential conflict of interest.
