# Supplementary material for: Understanding the Conductive Carbon Additive on Electrode/Electrolyte Interface Formation in Lithium-Ion Batteries via in situ Scanning Electrochemical Microscopy
Source: Front Chem. 2020 Feb 25;8:114. doi: 10.3389/fchem.2020.00114 (PMC7052374; doi:10.3389/fchem.2020.00114)
Supplement: Supplementary file 1 [file Data_Sheet_1.pdf]

Supporting Information

Understanding the Conductive Carbon Additive on Electrode/Electrolyte Interface Formation in Lithium-Ion Batteries via in situ Scanning Electrochemical Microscopy

Shuai Liu[1,2,†], Xiaojie Zeng[1,2,†], Dongqing Liu[1*], Shuwei Wang[1,2], Lihan Zhang[1,2], Rui Zhao[1,2], Feiyu Kang[1,2], Baohua Li[1*]

[1] Shenzhen Key Laboratory on Power Battery Safety Research and Shenzhen Geim Graphene Center, Graduate School at Shenzhen, Tsinghua University, Shenzhen 518055, China

[2] Laboratory of Advanced Materials, School of Materials Science and Engineering, Tsinghua University, Beijing 100084, China

***Correspondence**: Dongqing Liu liu.dongqing@sz.tsinghua.edu.cn

Baohua Li libh@mail.sz.tsinghua.edu.cn

[†] These authors have contributed equally to this work

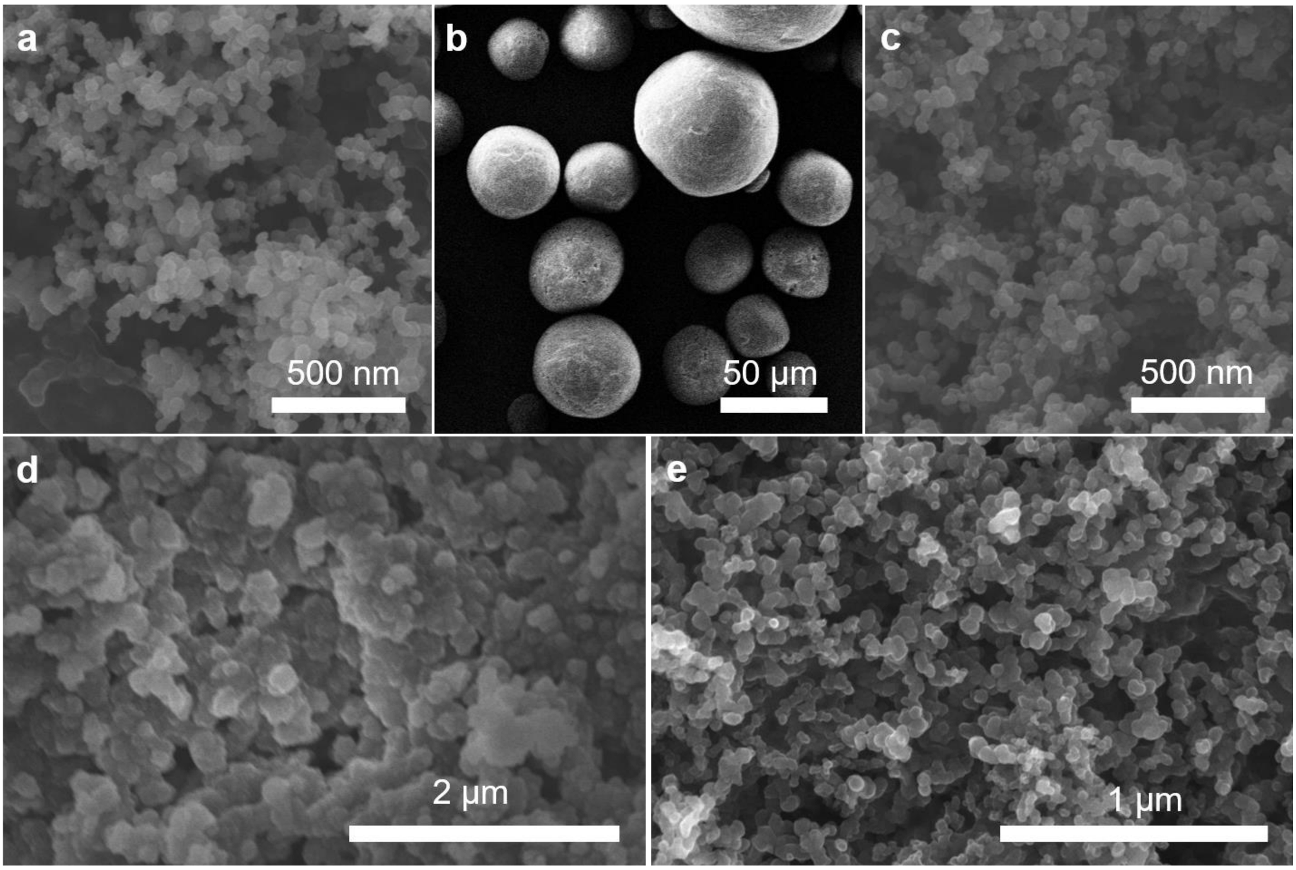

Figure S1. Scanning electron microscope of (a) Super-P powder (b) PVDF powder. Super-P electrode (c) before cycling, after cycles in the (d) low-potential region and (e) high-potential region.

Figure S2 show the feedback current area scans of the Super-P electrode at pristine state and the approach curve at pristine state. The approach curve is fitted with ExpDec function: $d=A\cdot\exp(-I/t)+d_o$ (d_o=162.1787, A=-7.2499E21, t=2.538E-10), thus the feedback current area scan could be converted to topography map as shown in Figure 2d.

The approach curve can be fitted into either $d = f(I)$ or $I = f(d)$ functions: $I = B \times \exp(d/t) + I_o$ (I_o= 1.17152E-8, B = 3.38835E-13, t = 20.88353), which can be used to determine the change of substrate topography on feedback current density. If we assume that the SEI with 50nm thickness uniformly distributed on the pristine electrode surface, then the average feedback current change induced by SEI determined via $I = f[d(x,y)+50nm] - f[d(x,y)]$, *~0.8 pA,* which is negligible compared with the nA scale feedback images.

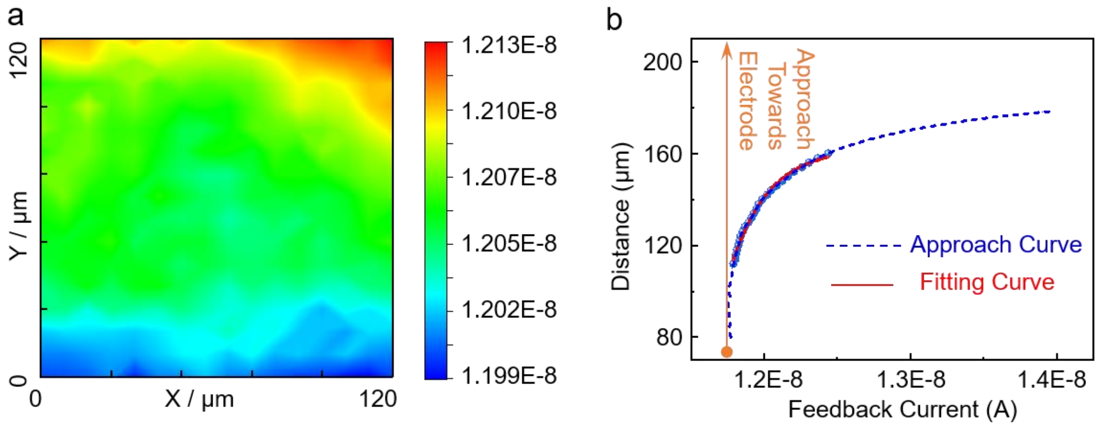

Figure S2. (a) SECM area scan of electrode at pristine state and (b) SECM probe approach curve towards pristine electrode.

After the 6th and 10th cycles in the low-potential region, the approach curve could be fitted into equations: I = exp(-18.71751+0.00168*d-0.00000500071*d^2) and I = exp(-19.46001+0.00674*d-0.0000262426*d^2), the feedback image (topography information and SEI passivation) and image induced solely by topography information are shown below:

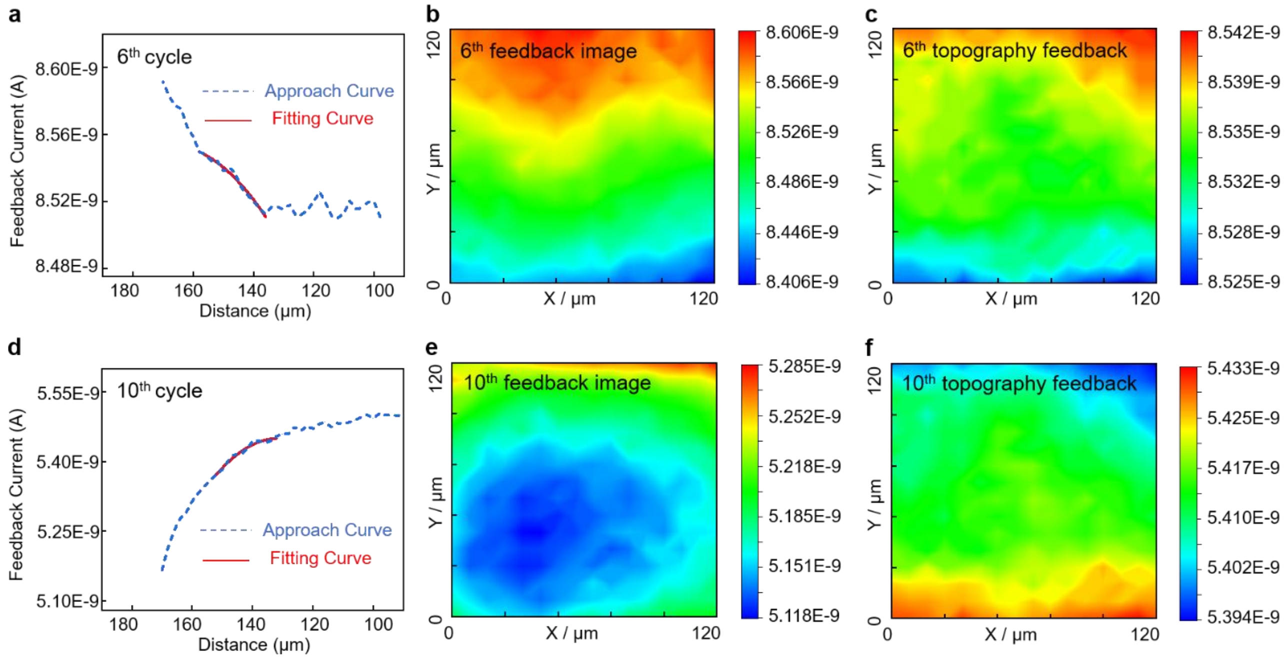

Figure S3. The approach curve, feedback image and topography feedback image for after the 6th cycle (a)(b)(c) and 10th cycle (d)(e)(f) obtained after cycles in the low-potential region.

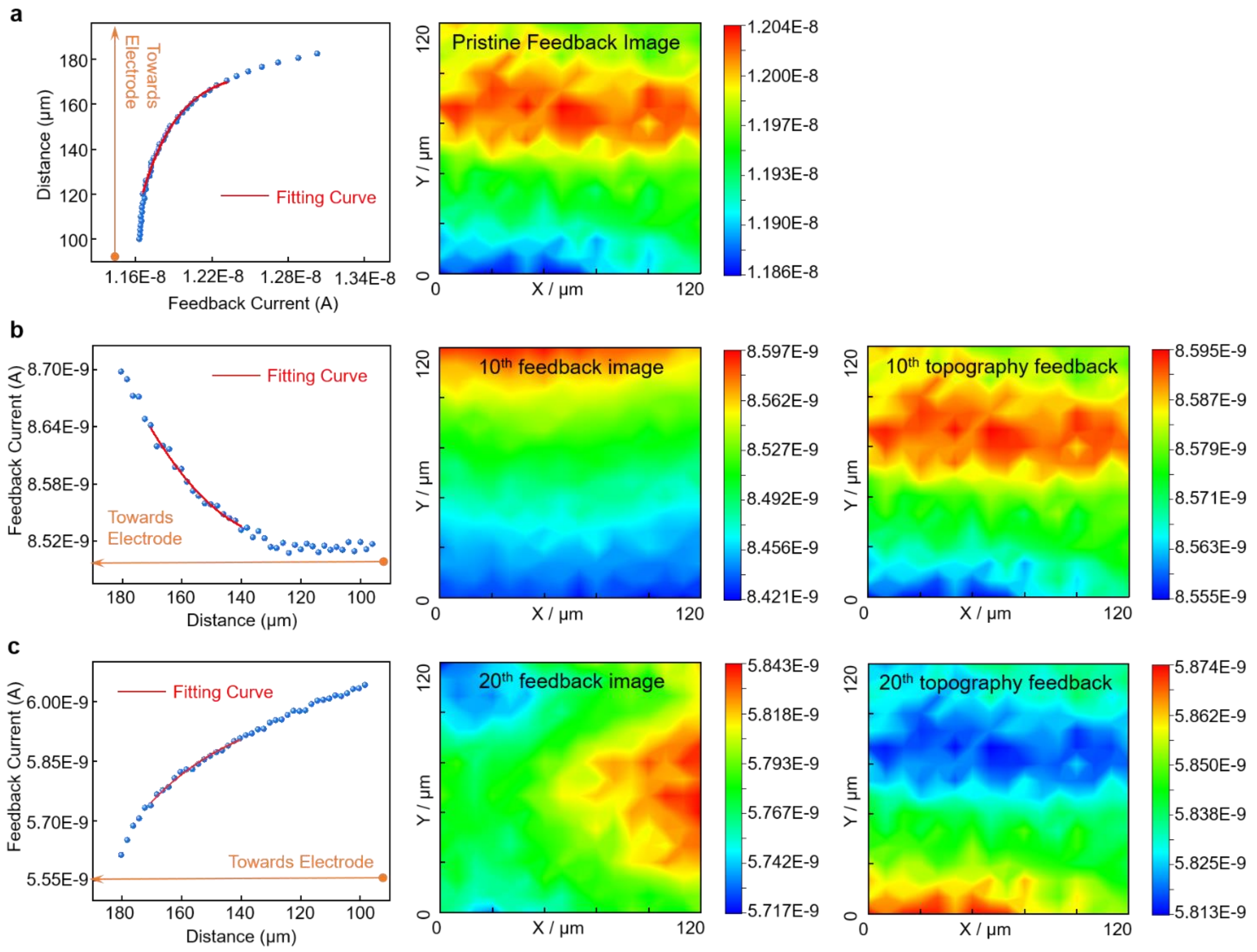

Figure S4. (a) SECM approach curve and area scan at pristine state. Approach curve, feedback image and topography feedback after (b) the 10th and (c) 20th cycles obtained with electrodes after cycles in the high-potential region.
